# Doxorubicin-induced cardiotoxicity is mediated by neutrophils through release of neutrophil elastase

**DOI:** 10.3389/fonc.2022.947604

**Published:** 2022-08-10

**Authors:** Anchit Bhagat, Pradeep Shrestha, Prince Jeyabal, Zhanglong Peng, Stephanie S. Watowich, Eugenie S. Kleinerman

**Affiliations:** ^1^ Department of Pediatrics, The University of Texas MD Anderson Cancer Center, Houston, TX, United States; ^2^ Department of Immunology, The University of Texas MD Anderson Cancer Center, Houston, TX, United States

**Keywords:** doxorubicin, neutrophils, cardio-oncology, neutrophil elastase inhibition, cardiotoxicity after chemotherapy

## Abstract

The mechanisms by which Doxorubicin (Dox) causes acute and late cardiotoxicity are not completely understood. One understudied area is the innate immune response, and in particular the role of neutrophils in Dox-induced cardiotoxicity. Here, using echocardiography, flow cytometry and immunofluorescence staining, we demonstrated increased infiltration of neutrophils that correlated with decreased heart function, disruption of vascular structures and increased collagen deposition in the heart after Dox treatment. Depleting neutrophils protected the heart from Dox-induced cardiotoxicity and changes in vascular structure. Furthermore, our data using neutrophil elastase (NE) knock-out mice and the NE inhibitor AZD9668 suggest that neutrophils cause this damage by releasing NE and that inhibiting NE can prevent Dox-induced cardiotoxicity. This work shows the role of neutrophils and NE in Doxorubicin-induced cardiotoxicity for the first time and suggests a new possible therapeutic intervention.

## Introduction

Anthracyclines are a class of chemotherapy drugs used to treat 50%-60% of childhood cancers including sarcomas (1). Some commonly used anthracyclines include Doxorubicin (Dox), mitoxantrone, epirubicin, idarubicin and daunorubicin. Dox, a derivative of daunorubicin, is one of the most effective chemotherapy agents against osteo- and Ewing sarcoma and cure rates correlate with the dose of Dox used. The 5-year survival rate for children younger than 16 years diagnosed with a malignancy is approximately 80%, a substantial improvement from that of <50% in the 1970s (2). To date, the anti-tumor activity of Dox has been shown to be mediated by i) DNA intercalation, resulting in tumor apoptosis; ii) inhibition of topoisomerase IIB and iii) generation of reactive oxygen species with subsequent cellular damage such as lipid peroxidation of cell membranes (3).

Despite the effectiveness of Dox, the incidence of treatment-related health complications in childhood cancer survivors (CCS) is high. One such complication is acute cardiac damage leading to a higher incidence of cardiovascular disease. Cardiotoxicity is defined by a reduction in left ventricular ejection fraction (LVEF): either global or specific to the interventricular septum. Cardiotoxicity can also be characterized as a reduction in LVEF from baseline to ≤ 55% in the presence of four signs or symptoms of heart failure (HF); or a reduction in LVEF ≥ 10% or an LVEF ≤ 55% without signs or symptoms of HF (3). LVEF, the chief measure of left ventricular systolic function, is the fraction of volume ejected during systole relative to the volume of blood in the ventricle at the end of diastole (4).

Cardiotoxicity can develop acutely or many years after completion of treatment. Indeed, up to 57% of CCS develop HF 30 years after diagnosis (5). In studies comparing CCS with their non-affected siblings, the lifetime incidence of heart disease was found to be five times higher (6, 7). It is speculated that the high risk of cardiac disease in CCS results from the acute damage to the juvenile heart cause by Dox, making the adult heart more vulnerable to stresses over time and predisposing to ischemic damage, myocardial infarction, valvular disease and other cardiomyopathies at an earlier age. Thus, a minor ischemic event that would result in little or no damage in a healthy individual may result in more substantial damage in a heart that has been damaged by Dox.

Additionally, Dox-induced cardiotoxicity is dose dependent. In patients who received more than 500 mg/m^2^ of anthracyclines, 18%-48% developed left ventricular dysfunction, in contrast to 3%-5% in those who received <500 mg/m^2^ (8, 9). Another study reported that patients who received a cumulative dose of 400 mg/m^2,^ had a 5% risk of developing heart failure which increased to 25% at 700 mg/m^2^ (7, 10, 11).

The mechanisms that contribute to Dox-induced acute and late cardiotoxicity are not completely understood. One understudied area is whether acute inflammation and the innate immune response contributes to Dox-induced cardiotoxicity. Other models of heart damage, such as myocardial infarction, showed that an inflammatory component was induced in response to damaged cardiomyocytes which included release of damage associated molecular patterns (DAMPs) that are detected by pattern recognition receptors (PRRs) (12). Neutrophils have been shown to play an important role in cardiac healing by clearing debris and necrotic tissue and inducing the polarization of monocytes/macrophages to the M2 phenotype- a critical process to healing. However, neutrophil persistence can be detrimental facilitating myocardial damage (13).

Neutrophils are the most abundant leukocytes and are the first to be recruited to an inflammatory site. Neutrophils act by releasing reactive oxygen species, a demonstrated mechanism of Dox-induced cardiotoxicity, as well as serine proteases. One such serine protease is neutrophil elastase (NE), which is secreted extracellularly from azurophilic granules upon activation (14). NE plays a crucial role in neutrophil mediated killing of bacteria. However, NE is also involved in tissue destruction and inflammation. NE release may therefore contribute to the cardiac tissue damage following Dox therapy. Studies have shown that NE has the capacity to degrade elastin, collagen and fibrinogen, thereby contributing to damage after myocardial infarction (MI). It has also been shown that NE deficiency improved survival and cardiac function post-MI (15). Neutrophils and NE-mediated tissue damage have been shown to play a role in arthritis and respiratory diseases (16, 17). However, whether neutrophils and NE contribute to Dox-induced acute cardiac damage has not been determined.

Here, we investigated whether neutrophils and NE contribute to cardiac damage and the acute and late cardiotoxicity that develops following Dox therapy.

## Materials and methods

### Mice

Our juvenile cardiotoxicity mouse model was used to determine the role of neutrophils in Dox-induced cardiotoxicity (18, 19). As Dox-induced cardiotoxicity has been shown to be increased in females, female 4-6 weeks old C57BL/6 mice were acquired from Experimental Radiation Oncology at MD Anderson Cancer Center (Houston, TX, USA). Female 4-6 weeks old Balb/c mice were acquired from Charles River Laboratory, Frederick. NE-deficient mice (NE^-/-^) of C57BL/6 background were kindly provided by Dr Stephanie Watowich. All mice were maintained in a pathogen-free animal facility and used in accordance with IACUC approved protocols. All experiments were performed in mice on Balb/c background, except for experiments involving neutrophil elastase, for which C57BL/6 mice were used.

### Echocardiography

Anaesthetized mice were assessed for cardiac function using transthoracic echocardiography (Vevo 3100 echocardiography with a 40MHz linear signal transducer and 550D probe; VisualSonics, Toronto, CA). M-mode short axis images were recorded at the level of the papillary muscles. The left ventricular (LV) muscle was bisected to obtain the optimal M-Mode(multimodal) selection. For each mouse, at least five B-mode and five M-mode images were recorded. All images were saved for analysis. Conventional echocardiographic measurements of the left ventricular function included ejection fraction (EF), fractional shortening (FS), end-diastolic diameter [LVED(d)], end-systolic dimension [LVID(s)], and anterior and posterior wall thickness. For long axis B-mode measurements, the endocardium was traced beginning from the mitral valve and excluding the papillary muscle. EF and FS were calculated by Vevo Lab software and is expressed as change from baseline measurement that is taken before Dox treatment.

### Neutrophil depletion

InVivoPlus anti-mouse Ly6G(IA8) (Bioxcell BP) antibody was used to deplete neutrophils. On days 2 and 9 of the experiment, anti-mouse Ly6G (500 µg) was administered intra-peritoneally. To confirm successful depletion blood samples were collected *via* retro-orbital bleeding. The red blood cells were lysed using ACK lysis buffer and subsequently washed with phosphate-buffered saline (PBS). The subsequent single cell suspension was incubated in PBS containing anti-mouse Ghost Violet Dye 510 (Tonbo Biosciences 13-0870) for 15-30 min at 4°C to identify dead cells. This was followed by incubation in PBS with 2% FBS (FACS buffer) containing FcR block for 10 minutes at 4°C. Subsequently, samples were stained with fluorescently conjugated antibodies against murine cell surface markers for 90 min at 4°C using the following reagents: anti-mouse CD45 PECy7 (Tonbo Biosciences), anti-mouse Ly6G FITC (BioLegend) and anti-mouse CD11b APC-Cy7(BioLegend). Stained single-cell suspensions were analyzed on a BD LSR Fortessa (BD Biosciences). Data analysis was performed using FlowJo v10 software (FlowJo, Ashland, OR, USA)

### Administration of doxorubicin *in vivo*

Doxorubicin from TEVA/Actavis(2mg/mL) was resuspended in PBS to make up to a total volume of 100 µL at a dosage of 2.5mg/kg. Resuspended Doxorubicin was administered to the mice intravenously *via* the tail vein twice a week for 2 weeks on days 4, 6, 11 and 13 as previously described (18, 19).

### Collection of heart sections

On day 14, 24h after the last dose of doxorubicin mice were euthanized and hearts were removed and split into two sections. One section was stored at -80°C (the optimal cutting temperature medium) to generate slides for immunofluorescence staining. The other section was chopped into small pieces (~2 mm) with a razor. Heart pieces were incubated in 2 mL Hanks’ Balanced Salt solution (HBSS). Type 2 collagenase (Worthington) was added at a dilution of 1:10 to the heart pieces in HBSS and incubated for 30 min in a shaking incubator at 37°C and 125 RPM. Digested cell suspensions were passed through 70 µm mesh filters; cells were subsequently washed with PBS. Following the wash, red blood cells were digested using ACK lysis buffer and then washed again with PBS in preparation for antibody staining.

### Immune profiling by antibody staining and flow cytometry

Single-cell suspensions were incubated in PBS containing anti-mouse Ghost Violet Dye 510 (Tonbo Biosciences) for 15-30 min at 4°C to identify dead cells. This is followed by incubation in PBS buffer with 2% FBS (FACS buffer) containing FcR block for 10 min at 4°C. The samples were the stained for 90 min at 4°C with the following antibodies against murine cell surface markers: anti-mouse CD45 PECy7 (Tonbo Biosciences), anti-mouse Ly6G FITC (BioLegend), anti-mouse F4/80 APC (eBioscience), anti-mouse CD11b APC-Cy7(BioLegend), anti-mouse Ly6C PerCP/Cy5.5(BioLegend). The stained single-cell suspensions were then analyzed using a BD LSR Fortessa (BD Biosciences). Data analysis was performed using FlowJo v10 software (FlowJo, Ashland, OR, USA).

### Real-time PCR

Quantitative real-time reverse transcription polymerase chain reaction (RT–PCR) was conducted to verify the changes in mRNA expressions. Extraction of the total RNA from cardiac tissues was performed utilizing the TRIzol (Invitrogen, MO, USA) reagent. The list of the primer sequences used in the study: mouse CXCL-1:(Forward:5’- ACCCGCTCGCTTCTCTGT-3), (Reverse: 5’- AAGGGAGCTTCAGGGTCAAG-3).

### Immunofluorescence staining

Frozen heart sections were fixed with acetone and then incubated with anti-mouse Ly6G antibody (Abcam), anti-mouse CD31 (BD Pharmingen) and anti-mouse NG2 (Santa Cruz Biotech. Fluorescence microscopy (Leica Microsystems) was used to analyze the slides. At least five different microscopy fields from different heart samples were examined using SimplePCI 6.0 software (Hamamatsu), and the average expression was quantified to determine relative expression.

### Masson Trichrome stain

Heart sections from mice were embedded in paraffin and then fixed in Bouin’s solution. Following fixation, sections were stained using the Sigma-Aldrich Trichrome Stain kit (Procedure No: - HT15), and images of stained slides taken using the Hamamatsu Nanozoomer. At least five different fields from different heart samples were analyzed using Leica Microsystems software (LAS X), and the average expression was quantified to determine relative expression.

### Bone marrow derived neutrophils

Femur and tibia from 4 to 6-week-old C57BL/6 control mice and NE-/- mice were collected and flushed with RPMI 1640 with 10% FBS and 1% penicillin/streptomycin. Histopaque 1119 and 1077 were used to create a density gradient to separate neutrophils from other immune cells. Purity of neutrophils was verified using flow cytometry.

### Neutrophil trans-well migration assay

The neutrophils were stained with CFSE (0.5 µM) dye and added to a 3 µm trans-well filter placed over the wells for 2 h. Transmigration was observed using an Incucyte system. Fluorescent intensity was quantified using Incucyte System S3 software. A chemoattractant for neutrophils: WKYMVm (conc: 100 nM) was used.

### TUNEL assay

Apoptotic cells were assessed using TUNEL staining with a DeadEnd Fluorometric TUNEL system (Promega) according to manufacturer’s instructions. Slides were fixed in 4% formaldehyde and then sections were incubated in TdT reaction mix for 1 h at 37° C in the dark. Slides were then rinsed thrice with PBS and observed under a fluorescence microscope. The number of apoptotic cells was determined by counting the cells that were positive for both green fluorescence and DAPI using ImageJ analysis software.

### Western blotting

Isolated hearts were homogenized, and the protein lysates obtained were run on a 7.5% polyacrylamide gel. The primary antibodies used for blotting included: cleaved caspase-3, caspase-3 and GAPDH (Cell Signaling Technology). Chemiluminescence was detected using ChemiDoc System (BioRad).

### NE inhibitor treatment

Mice were injected with AZD9668 intraperitoneally in vehicle(10%DMSO, 60%-PEG400, 30% Water) twice a day (100mg/kg) for the duration of Dox treatment (20).

### Statistics

Prism 8 software (GraphPad Software, San Diego, CA, USA) was used to perform statistical analyses. Data are shown as mean ± the standard error of the mean. An unpaired, two-tailed *t* test or Mann-Whitney test was used to compare two groups. A one-way ANOVA with Tukey comparison was performed to compare more than two groups. Differences were considered significant when *p*<0.05.

## Results

### Effect of Dox therapy on cardiac function and neutrophil infiltration

**Figure 1:**
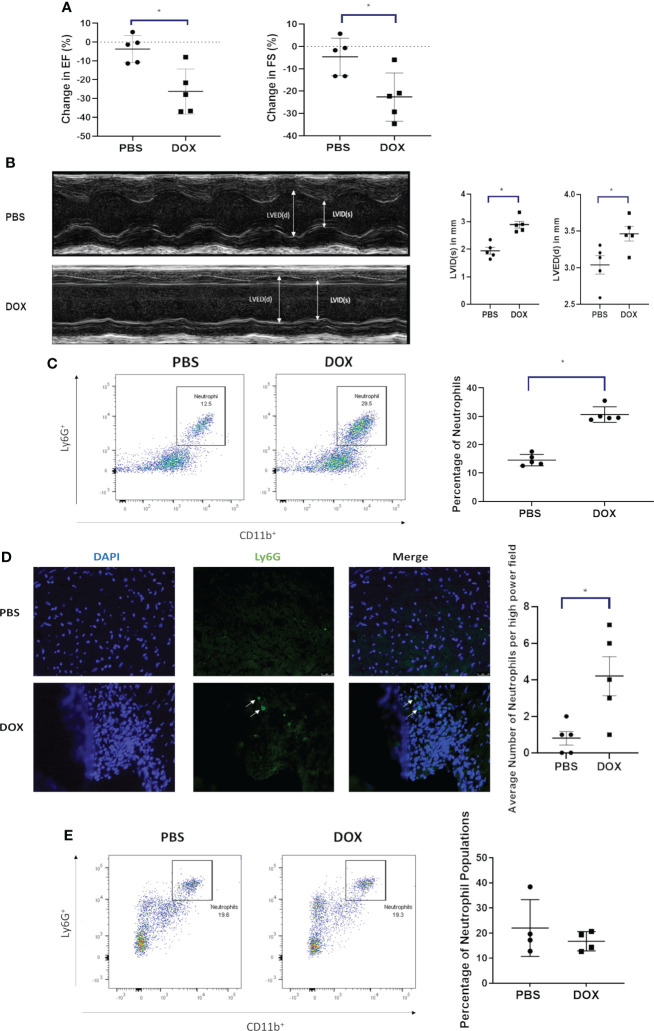
Effect of Doxorubicin (DOX) on heart function and neutrophil infiltration 24 h after therapy. **(A)** Ejection fraction (EF) and fractional shortening (FS) in mice were quantified by echocardiography 24 h after treatment with DOX or control phosphate-buffered saline (PBS); **(B)** Left ventricular internal dimension was measured in systole (LVID, s) and diastole (LVED,d). Data are presented as mean ± SEM, n=5 each, *p<0.05, a Mann-Whitney U-test was used to compare two groups.; **(C)** Infiltration of neutrophils (CD11b+ Ly6G+ cells) in hearts of mice treated with DOX or control phosphate-buffered saline(PBS) was quantified by flow cytometry 24 h after therapy; **(D)** Infiltration of neutrophils in hearts (Ly6G+) was quantified by immunofluorescence staining 24 h after therapy; **(E)** Infiltration of neutrophils (CD11b+ Ly6G+) in hearts was quantified by flow cytometry 11 weeks after therapy. Data are presented as mean ± SEM, n=5 each, *p<0.05, a Mann-Whitney U-test was used to compare two groups.

Our juvenile mouse cardiotoxicity model (18, 19) was used to investigate whether neutrophils contribute to Dox-induced cardiac damage. Echocardiograms showed a decrease in EF, FS and an increase in LVID(s) and LVED(d) 24 h after Dox therapy (**Figures 1A, B**). Heart tissue analyzed at the time by flow cytometry showed that neutrophils (CD11b^+^Ly6G^+^ cells, gated from the CD45^+^ population) were significantly elevated in the hearts of Dox-treated mice compared to controls (**Figure 1C**, p < 0.01). This finding was further validated by immunofluorescence staining using anti-Ly6G antibody (**Figure 1D**). Having demonstrated that neutrophils are increased 24 h after Dox treatment, we next determined whether neutrophils persisted in the heart. Mice were treated with Dox twice a week for 2 weeks and then evaluated for cardiac neutrophil infiltration 11 weeks after the therapy. No differences were observed in neutrophil numbers (CD11b^+^Ly6G^+^) between the hearts of Dox-treated and control mice 11 weeks after therapy (p=0.52) (**Figure 1E**). These data indicate that Dox therapy induced an acute increase in cardiac neutrophils, which was not sustained.

CXCL1 is involved in neutrophil recruitment. To determine if Dox-induced the upregulation of CXCL-1 in the heart tissue, CXCL1 mRNA was quantified and found to be significantly upregulated in the cardiac tissue 24 h after Dox therapy (**Supplemental Figure 1**, p<0.05).

### Effect of neutrophil depletion on *acute* Dox-induced cardiotoxicity

**Figure 2:**
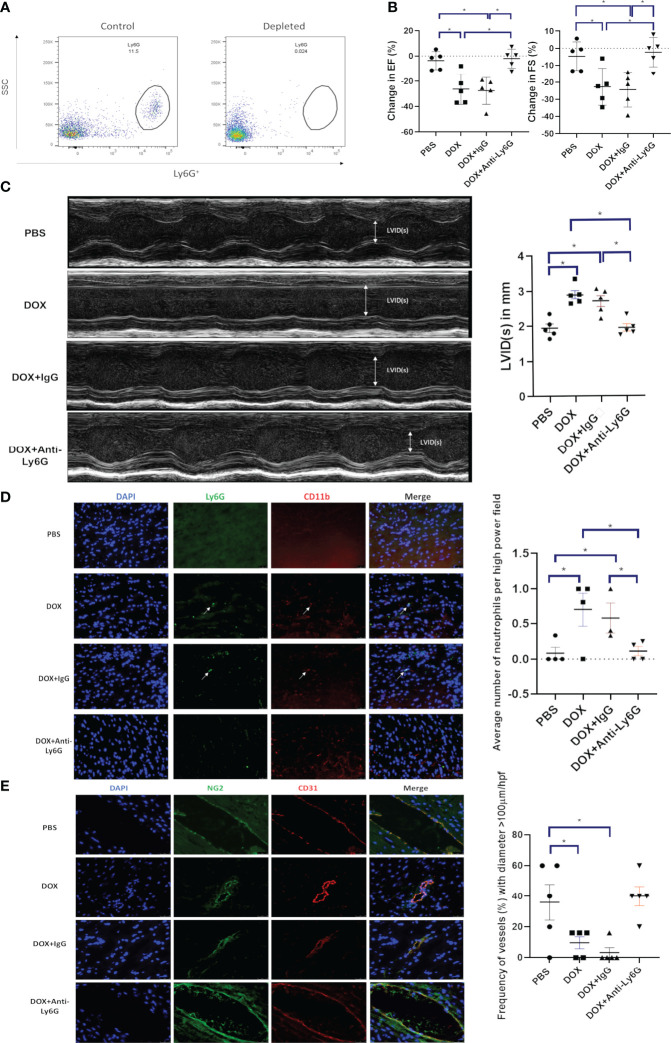
Effect of neutrophil depletion on heart function and vascular morphology 24 h after therapy. **(A)** Depletion of neutrophils was quantified by flow cytometry as described in Figure 1 b and c; **(B)** In mice with control phosphate-buffered saline(PBS), doxorubicin(DOX), DOX plus control IgG, or DOX plus anti-Ly6G antibody, ejection fraction (EF), fractional shortening (FS) were quantified; **(C)** Left ventricular internal dimension in systole (LVID(s)) were quantified; **(D)** Representative images of neutrophils (CD11b^+^ Ly6G^+^) in the hearts of mice treated with control phosphate-buffered saline(PBS), doxorubicin(DOX), DOX plus control IgG, or DOX plus anti-Ly6G; **(E)** Representative images of NG2^+^ and CD31^+^ vessels. Results are presented as mean ± SEM, n = 5 each, *p < 0.05, one-way ANOVA analysis with Tukey comparison was used to compare the groups.

We next determined whether depleting neutrophils using an anti-Ly6G antibody prior to Dox therapy inhibited the Dox-induced decrease in cardiac function as defined by EF, FS and LVID(s). Using Balb/c mice successful depletion of neutrophils was confirmed by flow cytometry in peripheral blood mononuclear cells (PBMCs) 24 h after the antibody treatment (**Figure 2A**). Twenty-four hours after Dox therapy the EF, FS and LVID(s) changes described above were seen in the Dox treated mice but not in the neutrophil-depleted mice (**Figures 2B, C**). Control IgG antibody given prior to Dox did not prevent Dox-induced cardiotoxicity. The decreases in EF and FS and increase in LVID(s) in the group treated with Dox and IgG was similar to that of the group treated with Dox only. Increased neutrophils were detected in heart sections of mice treated with Dox alone and Dox plus IgG (p<0.01). In contrast, neutrophils were not increased in hearts from mice treated with the anti-Ly6G antibody compared to control hearts (**Figure 2D**).

We previously demonstrated that there were changes in the morphology of the cardiac vessels 24 hours after Dox therapy (18). These included a decrease in CD31^+^ and NG2^+^ vessels, a decrease in the NG2^+^ pericyte coverage of the cardiac vessels and a decrease in the number of open lumen vessels with a diameter over 100 µm which correlated with a decrease in cardiac blood flow. Similar to our previous report, here we show that the cardiac vessels from mice treated with Dox alone or Dox plus IgG appeared more punctate and collapsed, a sign of decreased pericyte coverage. There was also a significant decrease in the number of vessels with open lumens >100 µm. These changes were not seen in the cardiac vessels from neutrophil depleted mice (**Figure 2E**). These data indicate that neutrophils contribute to damaging the cardiac vessels following Dox therapy, which leads to an acute effect on heart function. Depletion of neutrophils inhibited these Dox-induced structural and functional changes in heart.

### Effect of neutrophil depletion on *late* Dox-induced cardiotoxicity

**Figure 3:**
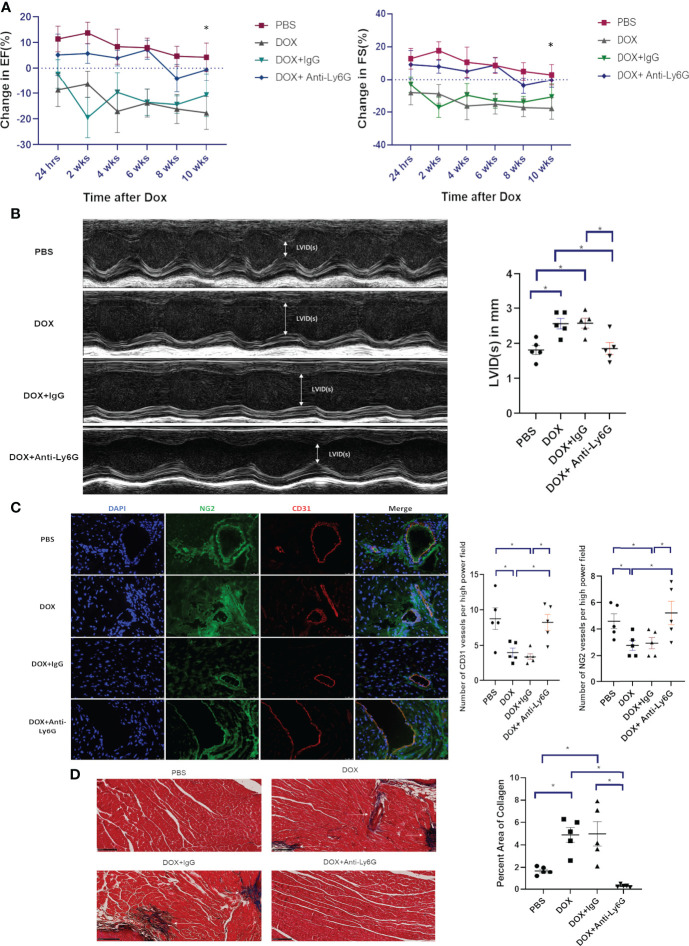
Effect of neutrophil depletion on heart function and vascular morphology 10 weeks after therapy. **(A)** In mice treated with control phosphate-buffered saline(PBS), doxorubicin(DOX), DOX plus control IgG, or DOX plus anti-Ly6G, ejection fraction(EF) and fractional shortening(FS) were quantified by echocardiography and followed for 10 weeks after therapy; **(B)** Left ventricular internal dimension in systole (LVID(s)) was also quantified 10 weeks after therapy; **(C)** Representative images and numbers of NG2^+^ and CD31^+^ vessels in the hearts of mice treated with control phosphate-buffered saline (PBS), doxorubicin (DOX), DOX plus control IgG, or DOX plus anti-Ly6G 10 weeks after therapy; **(D)** Collagen deposition in the hearts was determined using Masson trichrome staining 10 weeks after therapy completion. Data are presented as mean ± SEM, n = 5 each, *p < 0.05, one-way ANOVA analysis with Tukey comparison was used to compare the groups.

To determine whether neutrophil depletion decreased late cardiotoxicity, neutrophils were depleted as described above and mice were treated with Dox. Heart function was then monitored for 10 weeks after therapy. There was a continuous decline in EF and FS in Dox-treated mice (p<0.0001). In contrast, no change was seen in the neutrophil depleted mice treated with Dox (**Figure 3A**, p=0.3643). Additionally, LVID was increased in Dox and Dox plus IgG-treated mice compared to control mice 10 weeks after therapy. This was not seen in the neutrophil-depleted mice (**Figure 3B**). Ten weeks after therapy, the compromised vascular structures remained in the hearts from Dox and Dox plus IgG-treated mice, as defined by a decrease in CD31^+^ vessels and NG2^+^ vessels. By contrast, there remained, no significant decrease or difference in CD31^+^ or NG2^+^ vessels in hearts from neutrophil-depleted mice (**Figure 3C**).

We previously showed that while no cardiac fibrosis was seen 24 h after Dox treatment, there was significant cardiac fibrosis (collagen formation) 10-12 weeks after therapy (19). To determine whether neutrophil depletion impacted Dox-induced collagen formation, we performed Masson Trichrome staining 10 weeks after therapy. There was a significant increase in collagen in hearts from Dox-treated mice (p= 0.0015) but not in the hearts from neutrophil-depleted mice treated with Dox (**Figure 3D**, p=0.07).

Macrophages have been shown to contribute to cardiovascular diseases (21). We therefore determined whether our neutrophil depletion protocol was specific for neutrophils with no impact on macrophage numbers in the heart 24 h following Dox therapy. CD11b^+^Ly6Chi^+^ and CD11b^+^F4/80^+^ populations were used to identify and quantify pro-inflammatory monocytes and macrophages respectively. Monocyte and macrophage numbers were similar in the neutrophil- depleted and control mice treated with Dox (data not shown). This data indicates that early depletion of neutrophils prevented late stage cardiotoxicity from developing and also prevented excess collagen deposition. Additionally, we found that depletion of neutrophils did not affect the level of monocytes and macrophages in the cardiac microenvironment post Dox treatment.

### Contribution of neutrophil elastase to acute and late Dox-induced cardiotoxicity

**Figure 4:**
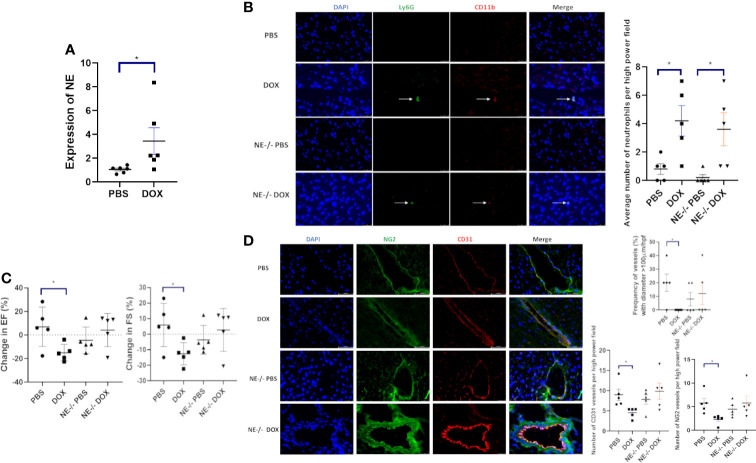
Effect of neutrophil elastase knockout (NE^-/-^) on heart function 24 h after therapy. **(A)** NE as measured by quantitative PCR in hearts of wild-type and NE^-/-^ mice 24 h after treatment with control phosphate-buffered saline(PBS) or doxorubicin(DOX); **(B)** Representative images of neutrophils (CD11b^+^ Ly6G^+^) 24 h after therapy; **(C)** Ejection Fraction (EF) and fractional shortening (FS) were quantified by echocardiography 24 h after therapy; **(D)** Effect of NE knockout on cardiac vessel morphology 24 h after therapy. Representative images, the number of NG2^+^ and CD31^+^ vessels, and the frequency (%) of vessels > 100µm/hpf 24h after therapy. Data are presented as mean ± SEM, n = 5 each, *p < 0.05, one-way ANOVA analysis with Tukey comparison was used to compare the groups.

Neutrophils release NE following activation. To investigate whether NE was elevated and contributed to Dox-induced cardiac damage, NE mRNA levels were quantified in the cardiac tissue of Dox treated and control mice 24 h after therapy. Higher NE mRNA levels were seen in the hearts from the Dox-treated mice (**Figure 4A**). NE^-/-^ mice were used to evaluate the contribution of NE to Dox-induced acute and late cardiotoxicity. First to confirm that neutrophils from the NE^-/-^ mice have similar migratory ability to neutrophils from wild type mice we performed an *in-vitro* trans-migration assay using WKYMVm, a chemoattractant for neutrophils. The migratory function of neutrophils from the NE^-/-^ mice was not significantly different compared to neutrophils from wild type mice (**Supplemental Figure 2**). Next, we confirmed that Dox therapy induced an increase in cardiac neutrophils 24 h after therapy in these NE^-/-^ mice. Similar to wild type mice neutrophils were significantly elevated in Dox-treated NE^-/-^ mice (**Figure 4B**, p=0.04). Therefore, the NE knockout did not inhibit neutrophil migration into the heart 24 h after Dox treatment. There was a significant decrease in EF and FS in the Dox-treated wild-type mice but not in the NE^-/-^treated with Dox. The EF and FS in Dox-treated NE^-/-^ mice were similar to those of NE^-/-^ control mice (**Figure 4C**). Blood vessel morphology (as determined by quantifying the number of open vessels with diameter >100µm) was also not significantly different in the NE^-/-^ control and NE^-/-^ Dox treated mice and there was no decrease in CD31^+^ and NG2^+^ vessels (**Figure 4D**, p=0.0115).

**Figure 5:**
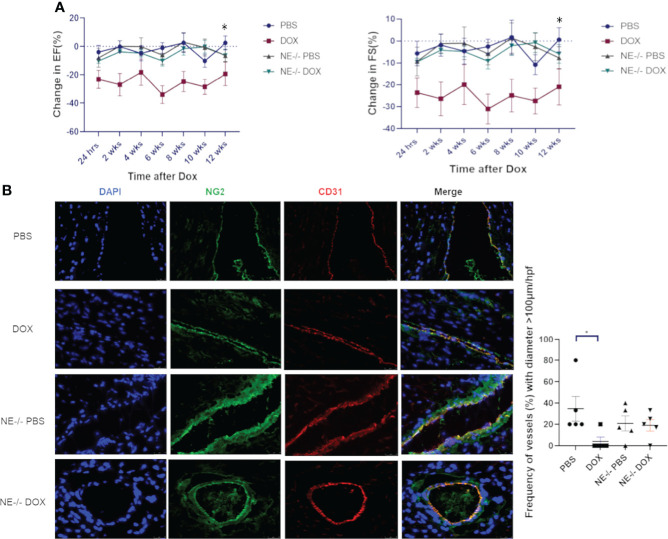
Effect of neutrophil elastase knockout (NE^-/-^) on heart function and cardiac vessel morphology 12 weeks after therapy. **(A)** Ejection Fraction (EF) and fractional shortening (FS) were quantified by echocardiography and followed for 12 weeks after treatment with control phosphate-buffered saline (PBS) or doxorubicin (DOX) in wild-type and NE^-/-^ mice; **(B)** Representative images of cardiac NG2^+^ and CD31^+^ vessels 12 weeks after therapy and frequency (%) vessels with >100µm/hpf. Data are presented as mean ± SEM, n = 5 each, *p < 0.05, one-way ANOVA analysis with Tukey comparison was used to compare the groups.

To evaluate the role of NE in the late stage of Dox-induced cardiotoxicity mice were treated with Dox for 2 weeks, and cardiac function monitored for 12 weeks. There was a significant decrease in EF and FS in the Dox-treated wild type mice 24 h after therapy that persisted over 12 weeks. In contrast, no decrease in EF or FS was seen in the Dox treated NE^-/-^ mice (**Figure 5A**). Cardiac vascular morphology was also unchanged in the Dox-treated NE^-/-^ mice (**Figure 5B**). The number of open vessels with a diameter >100µm in the NE^-/-^ control and NE^-/-^ Dox treated mice were not significantly different (**Figure 5B**). This data indicated that inhibiting or blocking NE prevented both the acute and late Dox-induced cardiac damage.

**Figure 6:**
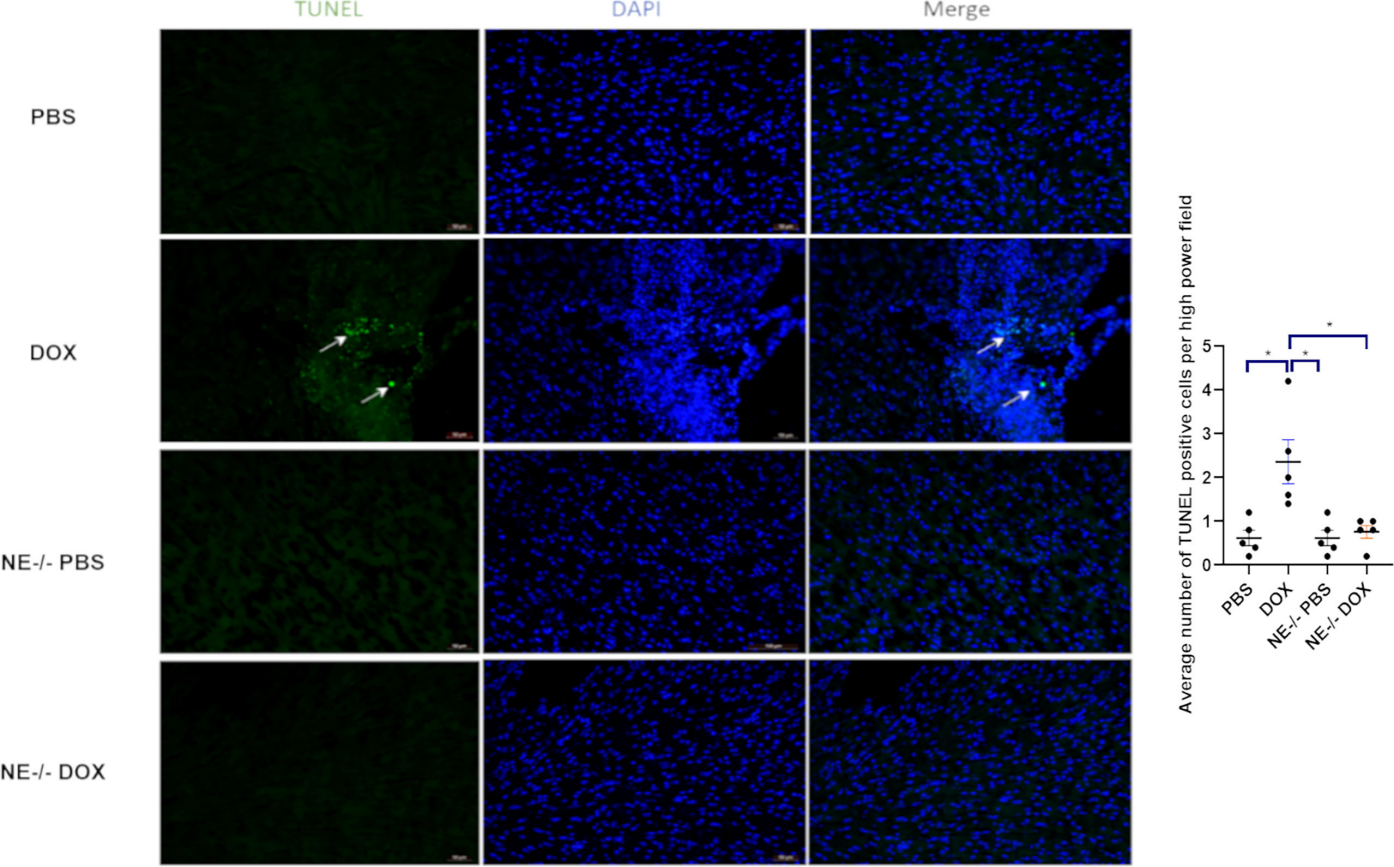
Effect of neutrophil elastase knockout (NE^-/-^) on apoptosis in heart tissue following doxorubicin (Dox) treatment. Representative TUNEL staining images of heart sections 24 h after Dox treatment; Data are presented as mean ± SEM, n = 5 each, *p < 0.05, one-way ANOVA analysis with Tukey comparison was used to compare the groups.

Ogura et al. showed that the NE caused cardiomyocyte apoptosis in myocardial infarction (22). We wanted to investigate these findings in our context of Dox-induced cardiotoxicity. TUNEL staining was used to identify apoptotic cells. The number of TUNEL cells was significantly higher in Dox treated mice as compared to NE^-/-^ mice (**Figure 6**). We also found that the TUNEL positive cells were specific to cardiomyocytes as evidenced by staining with cardiac troponin (**Supplemental Figure 3**). Additionally, we quantified cleaved caspase-3 levels in Dox-treated control and NE^-/-^ mice to investigate the effect of decreased NE on cardiomyocyte apoptosis after Dox treatment. Cleaved caspase-3 protein was significantly elevated in the control Dox treated hearts as compared to the NE^-/-^ Dox treated hearts (**Supplemental Figure 4**).

**Figure 7:**
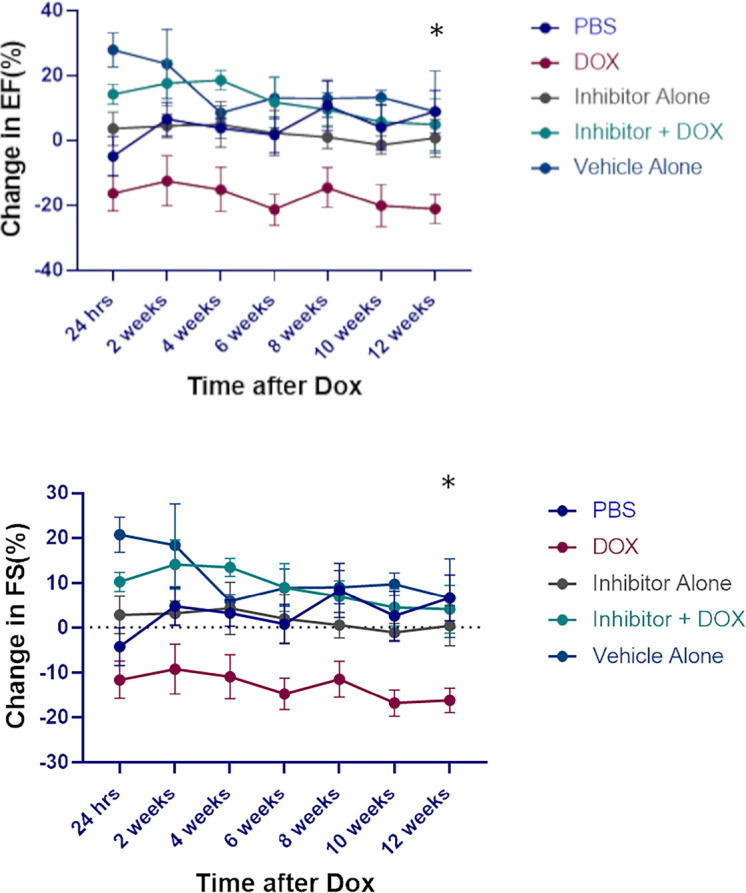
Effect of neutrophil elastase (NE) inhibitor on heart tissue following doxorubicin (DOX) treatment. Ejection fraction (EF) and fractional shortening (FS) were quantified by echocardiography up to 12 weeks after therapy. Data are presented as mean ± SEM, n = 5 each, *p < 0.05, one-way ANOVA analysis with Tukey comparison was used to compare the groups.

Having demonstrated that NE contributed to Dox-induced cardiotoxicity we investigated the therapeutic potential of an NE inhibitor, AZD9668. Here mice were treated with Dox, AZD9668 or both drugs concurrently. Decreased EF and FS were seen in the Dox-treated mice but not in mice that received Dox plus AZD9668 (**Figure 7**). This effect persisted for 12 weeks after Dox treatment had ended indicating that an NE inhibitor has therapeutic potential to prevent Dox-induced heart damage.

## Discussion

Dox continues to be an integral part of treatment for pediatric and adolescent and young adult patients (AYA) with sarcoma due to the lack of new effective targeted therapies, including immunotherapies. Unfortunately, Dox causes acute damage to the heart resulting in late cardiac morbidities which range from changes in myocardial structure and function, to severe cardiomyopathy, valvular disease and congestive HF in long-term survivors. These cardiac morbidities compromise quality of life (QOL) and longevity and may result in a need for cardiac transplant (23, 24). Indeed, Dox-induced cardiotoxicity is the second leading cause of death in childhood and AYA cancer survivors (25, 26). Therefore, strategies to limit the acute heart damage caused by Dox are expected to result in lower cardiac morbidities, improved longevity and better QOL. Identifying effective interventions requires an understanding of the *multiple* mechanisms that contribute to Dox-induced cardiotoxicity (27).

Here we demonstrated for the first time that Dox therapy induced the acute infiltration of neutrophils into the heart (as quantified by flow cytometry and immunofluorescence staining; (**Figure 2**). This was accompanied by an acute decline in cardiac function, (defined by decreased in EF and FS and increased LVID), an alteration in cardiac vascular morphology, and a decrease in the number of cardiac vessels with open lumens (**Figures 1**, **2**). The increase in cardiac neutrophils did not persist. No differences in neutrophil numbers in the heart were seen 4-11 weeks after therapy in Dox-treated versus control mice. This is in accordance with previous studies demonstrating that neutrophil infiltration was short lived, peaked early but dissipated at later time points (16). Despite the normalization of neutrophil numbers, the decreased cardiac function persisted for 2-12 weeks after therapy (**Figure 3**).

The importance of neutrophils and NE in the acute Dox-induced cardiotoxicity process was confirmed using neutrophil-depletion techniques and NE-knockout mice. We confirmed that neutrophil depletion using anti-Ly6G was successful and that this resulted in no increase in cardiac neutrophils 24h following Dox therapy (**Figure 2**). Our data showed that Dox-induced acute cardiotoxicity was not seen in either neutrophil-depleted mice or NE^-/-^ mice. Under these conditions EF, FS, LVID(s) and vascular morphology were unchanged 24 h after Dox therapy. We also confirmed that the increase in neutrophil infiltration following Dox therapy in the NE^-/-^ mice was not significantly different than that seen in wild-type mice, indicating that the absence of NE did not interfere with the neutrophils’ ability to migrate into the heart (**Figure 3**). Similar to our results, Hirche et al. showed that during infection the neutrophil recruitment to sites of inflammation were not hindered in elastase knockout mice (28). Their data showed no differences in the ability of WT and NE^−/−^ neutrophils to migrate to sites of inflammation. This is in contrast to the findings of Voisin et al. where impaired neutrophil invasion into the heart tissue was seen following ischemia/reperfusion injury (29). The difference between our findings may be due to the fact that we are investigating Dox-induced cardiac damage which is not the same as damage induced by ischemia.

We have previously shown that Dox therapy acutely affected cardiac vascular structure, morphology and function as defined by decreased pericytes, collapsed cardiac vessels, and a decrease in the number of vessels with open lumens, resulting in decreased cardiac diastolic and systolic blood flow (18, 19). Neutrophil-depleted and NE^-/-^ mice showed none of these acute changes in vascular structure 24h after Dox therapy. We interpret this to mean that neutrophil depletion and the absence of NE prevented acute Dox-Induced vascular damage.

Cardiotoxicity is known to develop in survivors many years after treatment. We therefore investigated whether neutrophil depletion and NE knockout prevented the late Dox-induced cardiotoxicity. When we evaluated heart function 10 weeks after treatment was completed, we observed significant decreases in EF and FS, and an increase in LVID(s) in the Dox-treated mice. However, as demonstrated in the acute cardiotoxicity experiments, there was no change in cardiac function in the neutrophil depleted mice. The vascular changes also were not observed in the neutrophil- depleted mice. In addition, there was significant collagen deposition consistent with the development of cardiac fibrosis in the hearts of the Dox-treated control mice but not the neutrophil-depleted mice treated with Dox.

Taken together, these data further confirm that neutrophils contribute to the acute heart damage caused by Dox. This acute damage had a prolonged effect on the heart. Inhibiting this acute phase prevented the development of late-stage cardiotoxicity. Our data suggest that neutrophils contribute to both acute and late Dox-induced cardiotoxicity. Neutrophil depletion had no effect on macrophage infiltration following Dox therapy. We also demonstrated in the heart tissue that after Dox treatment Dox there was increased expression of CXCL1, a cytokine involved in neutrophil recruitment. These results suggest a direct mechanism by which Dox induces neutrophil infiltration into the heart.

Previous reports have also shown that neutrophils are recruited to the heart following cardiac injury such as myocardial infarction. Here damaged cardiomyocytes act as DAMPs that are detected by PRRs which release cytokines such as CXCL1 that helps recruit neutrophils (12). Furthermore, when we performed TUNEL assay on wild-type and NE^-/-^ mice following Dox treatment we observed a significant number of apoptotic cells in hearts from the wild-type mice but not the NE^-/-^ mice. Taken together, these results indicate that neutrophils through the release of NE contribute to cardiotoxicity and that inhibiting NE prevents this cardiotoxicity. To test the therapeutic potential of targeting NE we administered the NE inhibitor AZD9668 during Dox treatment. We found that the EF and FS did not change in mice treated with AZD9668 and Dox and more importantly that this protection persisted for up to 12 weeks after the Dox treatment had been completed. These results suggest that targeting NE during Dox therapy may decrease acute Dox-induced cardiac damage.

In this study we focused on NE as we have observed higher expression levels of this enzyme in hearts with Dox. However, the role of other serine proteases that neutrophils release upon degranulation such as cathepsin G and proteinase 3 (30), may also contribute to Dox-induced cardiotoxicity and should be investigated. Additionally, we briefly monitored levels of another myeloid cell population, monocytes/macrophages, in hearts of Dox-treated mice after neutrophil depletion. Traditionally, macrophages participate in phagocytosis, chemotaxis, secretion and antigen presentation for immune defense and tissue healing (31). The plastic nature of these cells has rendered their exact function in the cardiac microenvironment post heart damage unclear (32, 33). Hence, a deeper look into the role of macrophages with further characterization of macrophages that are characterized into either M1(classically activated pro-inflammatory) or M2 (alternately activated anti-inflammatory) (34, 35) in Dox-induced heart damage needs to be done.

**Figure 8:**
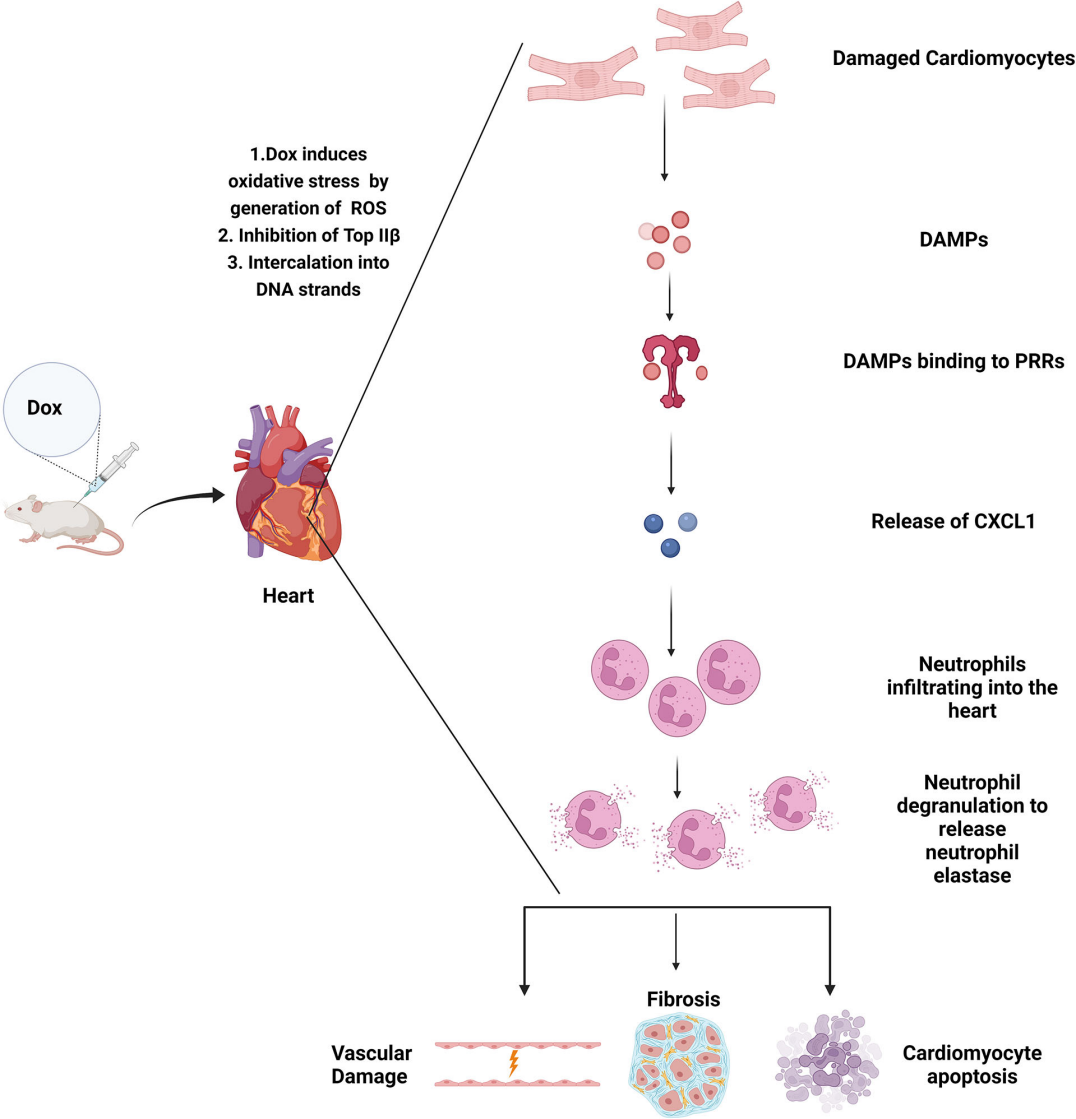
Graphical Abstract.

In summary our results show that neutrophils contribute to Dox-induced cardiac damage, through the release of NE leading to vascular damage and decreased heart function that persist many weeks after therapy completion (**Figure 8**). This is the first study of its kind to demonstrate that neutrophils and neutrophil elastase are involved in Dox-induced cardiotoxicity. In pre-clinical models in rats, NE inhibitors ablated the ischemia-induced myocardial damage and coronary endothelial dysfunction (36). Furthermore in clinical trials, NE inhibitors have been used to treat cystic fibrosis and chronic obstructive pulmonary disease and have been found to be safe and well tolerated and effective in curbing the excess inflammatory response (37, 38). AZD9668 in particular, has been used in clinical trials for patients with chronic obstructive airway disease and was found to have no significant toxicity while showing promising therapeutic potential in early phase studies (39). In our cardiotoxicity mouse model, AZD9668 was well tolerated and effective in inhibiting Dox-induced cardiac damage and in preserving heart function after Dox therapy. This is a significant finding with translational potential to decrease the incidence and degree of cardiomyopathies in CCS, which in turn will impact both QOL and patient longevity, as cardiac disease is the second leading cause of death in these individuals (40). Our data supports consideration for the inclusion of a NE inhibitor with Dox with the goal of preventing Dox-induced acute and late cardiotoxicity in survivors. NE inhibitors may decrease the acute inflammatory response induced by Dox preventing cardiomyocyte apoptosis and the late fibrosis that develops.

## Conclusion

In conclusion we found that neutrophils play an important role in Dox-induced cardiotoxicity through release of neutrophil elastase. Our finds suggest a new treatment approach for mitigating this damage during Dox treatment.

## Data availability statement

The datasets presented in this study can be found in online repositories. The names of the repository/repositories and accession number(s) can be found in the article/**Supplementary Material**.

## Ethics statement

This study was reviewed and approved by IACUC MD Anderson Cancer Center.

## Author contributions

EK was involved in experimental designs, data analysis and revision of manuscript. SW was involved in experimental design, manuscript revision and provided NE-/- mice. AB was involved in experimental design, performed experiments, acquired and analyzed the data, prepared figures and wrote the manuscript. PS was involved in experimental design, performed experiments and data analysis. PJ and ZP helped provide heart tissue samples for analysis. All authors contributed to the article and approved the submitted version.

## Funding

This work was supported in part by the Mary V. and John A. Reilly Distinguished Chair Fund, National Cancer Institute P30CA016672 institutional core grant and a grant from the Cancer Prevention and Research Institute of Texas (RP200381).

## Acknowledgments

We thank Dr Khandan Keyomarsi, Amriti Lulla and Christopher Carroll at The University of Texas MD Anderson Cancer Center for their assistance with purchasing the NE inhibitor AZD9668. We also thank Fei Wang and Yuanzheng Yang for assistance and advice in designing some experiments. We would also like to thank Bryan Tutt and Scientific Publications Service, Research Medical Library for their assistance in editing this manuscript. Pradeep Shrestha PhD is an Odyssey Fellow and is supported by the Odyssey Program and HEB Corporation at the University of Texas MD Anderson Cancer Center.

## Conflict of interest

The authors declare that the research was conducted in the absence of any commercial or financial relationships that could be construed as a potential conflict of interest.

## Publisher’s note

All claims expressed in this article are solely those of the authors and do not necessarily represent those of their affiliated organizations, or those of the publisher, the editors and the reviewers. Any product that may be evaluated in this article, or claim that may be made by its manufacturer, is not guaranteed or endorsed by the publisher.

## Supplementary material

Supplementary Figure 1 CXCL1 expression in cardiac tissue as quantified by qPCR 24 h after Dox treatment. Data are presented as mean ± SEM, n = 4 each, **p* < 0.05, a Mann-Whitney U-test was used to compare two groups.

Supplementary Figure 2 Neutrophil Transmigration following doxorubicin treatment was quantified by Incucyte in the presence and absence of a chemoattractant, a Mann-Whitney U-test was used to compare two groups.

Supplementary Figure 3 Representative TUNEL staining images of heart sections 24 h after Dox treatment counterstained with CnTI (Cardiac Troponin I).

Supplementary Figure 4 Representative images of Western blots for caspase-3, cleaved caspase-3 and GAPDH from hearts 24 h after treatment; the protein levels of cleaved caspase-3 were quantified using densitometry analysis and normalized to the levels of caspase-3; Data are presented as mean ± SEM, n = 3 each, a Mann-Whitney U-test was used to compare two groups.
