# Supplementary figures and images for: Doxorubicin-induced cardiotoxicity is mediated by neutrophils through release of neutrophil elastase
Source: Front Oncol. 2022 Aug 10;12:947604. doi: 10.3389/fonc.2022.947604 (PMC9400062; doi:10.3389/fonc.2022.947604)

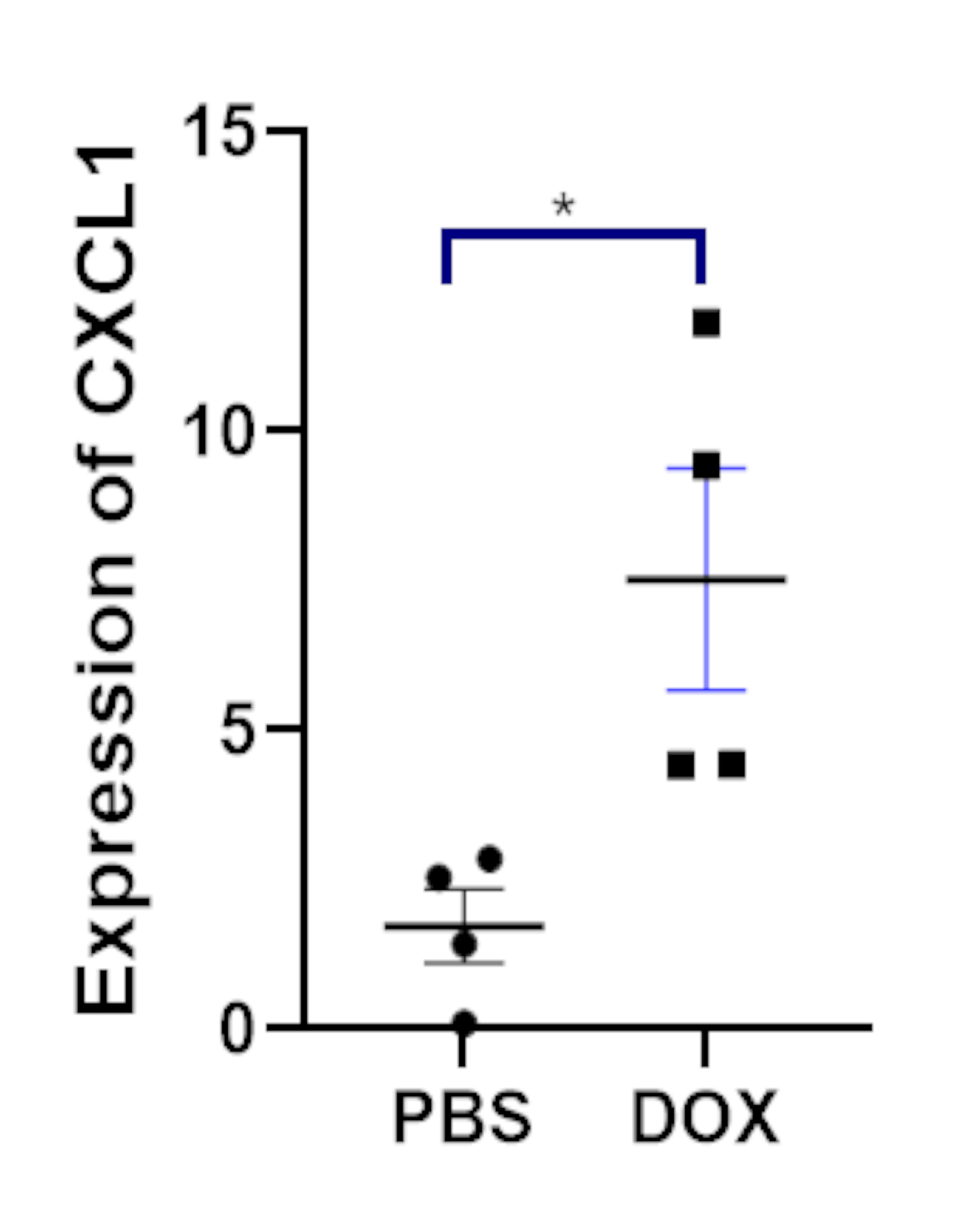

Supplement: Supplementary Figure 1 — CXCL1 expression in cardiac tissue as quantified by qPCR 24 h after Dox treatment. Data are presented as mean ± SEM, n = 4 each, *p < 0.05, a Mann-Whitney U-test was used to compare two groups. [file Image_1.tif]

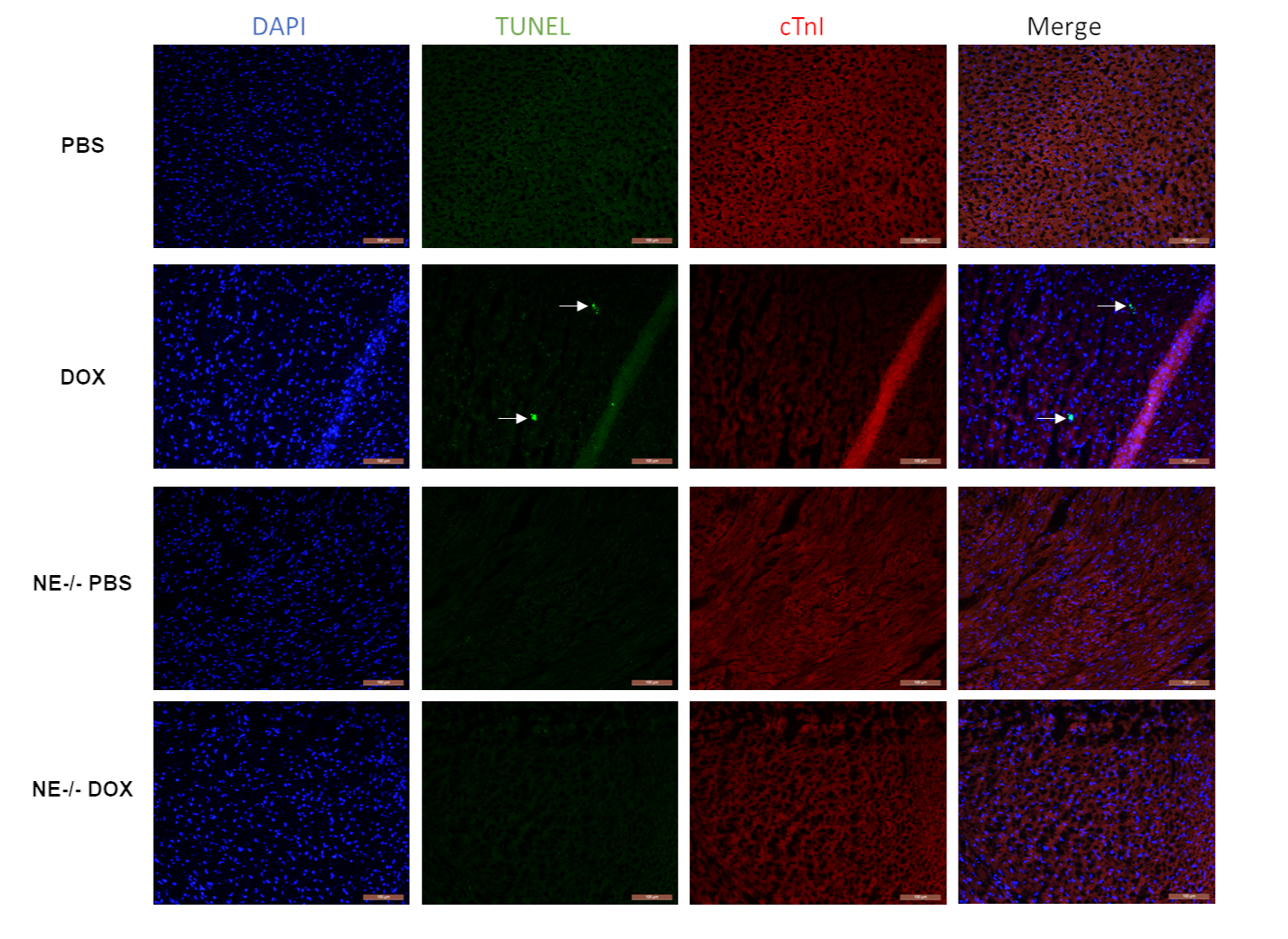

Supplement: Supplementary Figure 3 — Representative TUNEL staining images of heart sections 24 h after Dox treatment counterstained with CnTI (Cardiac Troponin I). [file Image_3.tif]

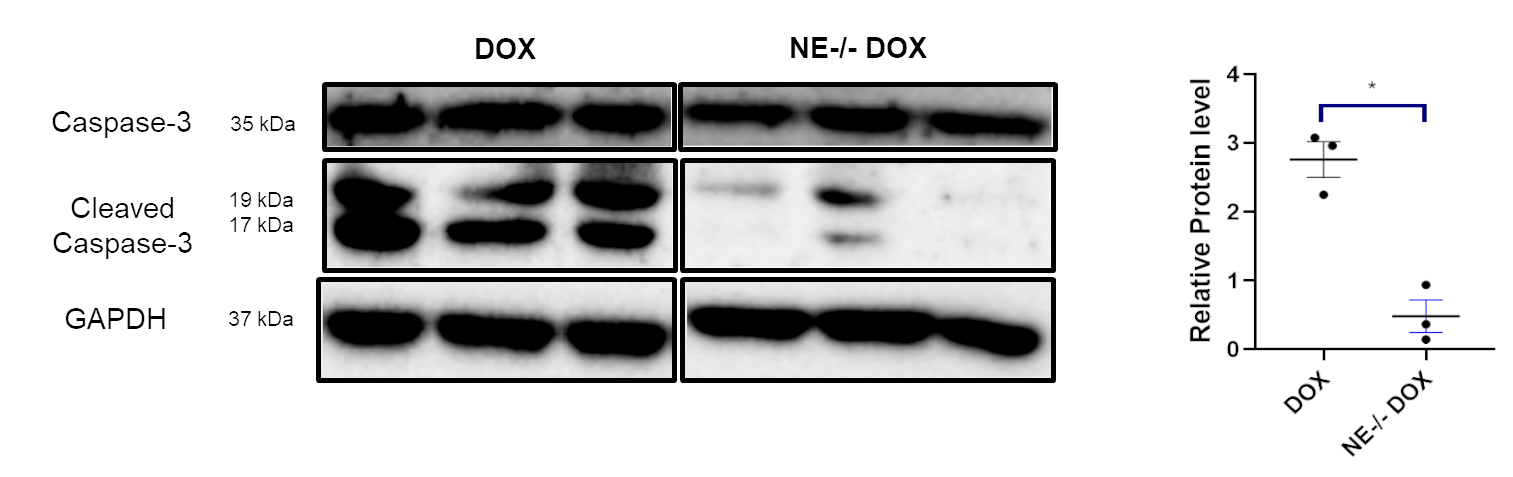

Supplement: Supplementary Figure 4 — Representative images of Western blots for caspase-3, cleaved caspase-3 and GAPDH from hearts 24 h after treatment; the protein levels of cleaved caspase-3 were quantified using densitometry analysis and normalized to the levels of caspase-3; Data are presented as mean ± SEM, n = 3 each, a Mann-Whitney U-test was used to compare two groups. [file Image_4.tif]
